# New learning technique based on real-time kinematic feedback from an inertial sensor for manual therapy in shoulder joint: a randomised trial

**DOI:** 10.1186/s12909-024-05649-y

**Published:** 2024-09-11

**Authors:** Manuel Trinidad-Fernández, Francisco González-Molina, Cristina Roldán-Jiménez, Peter Vaes, Manuel González-Sánchez, Antonio Ignacio Cuesta-Vargas

**Affiliations:** 1 Departamento de Fisioterapia, Universidad de Málaga, Málaga, Spain; 2 Grupo de Investigación Clinimetría F-14, Instituto de Investigación Biomédica de Málaga y Plataforma en Nanomedicina (IBIMA-Plataforma Bionand), Málaga, Spain; 3 Rehabilitation Research (RERE) Research Group, Vrije Universiteit Brussel, Brussels, Belgium

**Keywords:** Education, Manual therapies, Training, Feedback

## Abstract

**Background:**

Reducing teacher subjectivity and checking skill corrections have an impact on the manual therapy learning, one of the most crucial components of physical therapy clinical practise. The aim of this study was to analyse the effectiveness of a kinematic real-time feedback strategy (KRTF) with an inertial sensor as a new methodology for the learning of glenohumeral joint mobilisation, comparing it with the traditional teaching method.

**Methods:**

This study was a randomised trial. 59 undergraduate students without experience in manual therapy were randomised into two different groups (G1: Traditional methods group; G2: KRTF group). G1: students would practice the technique while an expert in manual therapy would supervise them. G2: could perform the mobilisation and observe the kinematic characteristics of the technique on a laptop. For the two movements that compose the mobilisation (angulation and translation), the result variables extracted were: maximum displacement, minimum displacement, area under the curve and the difference between the area under the curve of angulation and translation. In addition, the consistency of the measurement and reliability were calculated, too.

**Results:**

Some significant differences were observed within groups, between groups and in the group x time interaction, the difference between the angulation and translation area. The synchronization of the movements in in the post comparison was better in G2 because the differences in the areas of both movements were significantly smaller (Mean Difference G1 vs. G2 = 1111.4°s (*p* > 0.05)).

**Conclusions:**

After comparing the kinematic variables recorded between the two intervention groups analysed in the present study, we observed that the kinematic registers were significantly different between the two groups, with a higher evolution in the KRTF group compared to the traditional learning method. The effectiveness of KRTF was proved over the traditional teaching methods in facilitating the learning process of the glenohumeral joint mobilisation.

**ClinicalTrials.gov ID:**

NCT02504710, 22/07/2015.

## Background

**Fig. 1:**
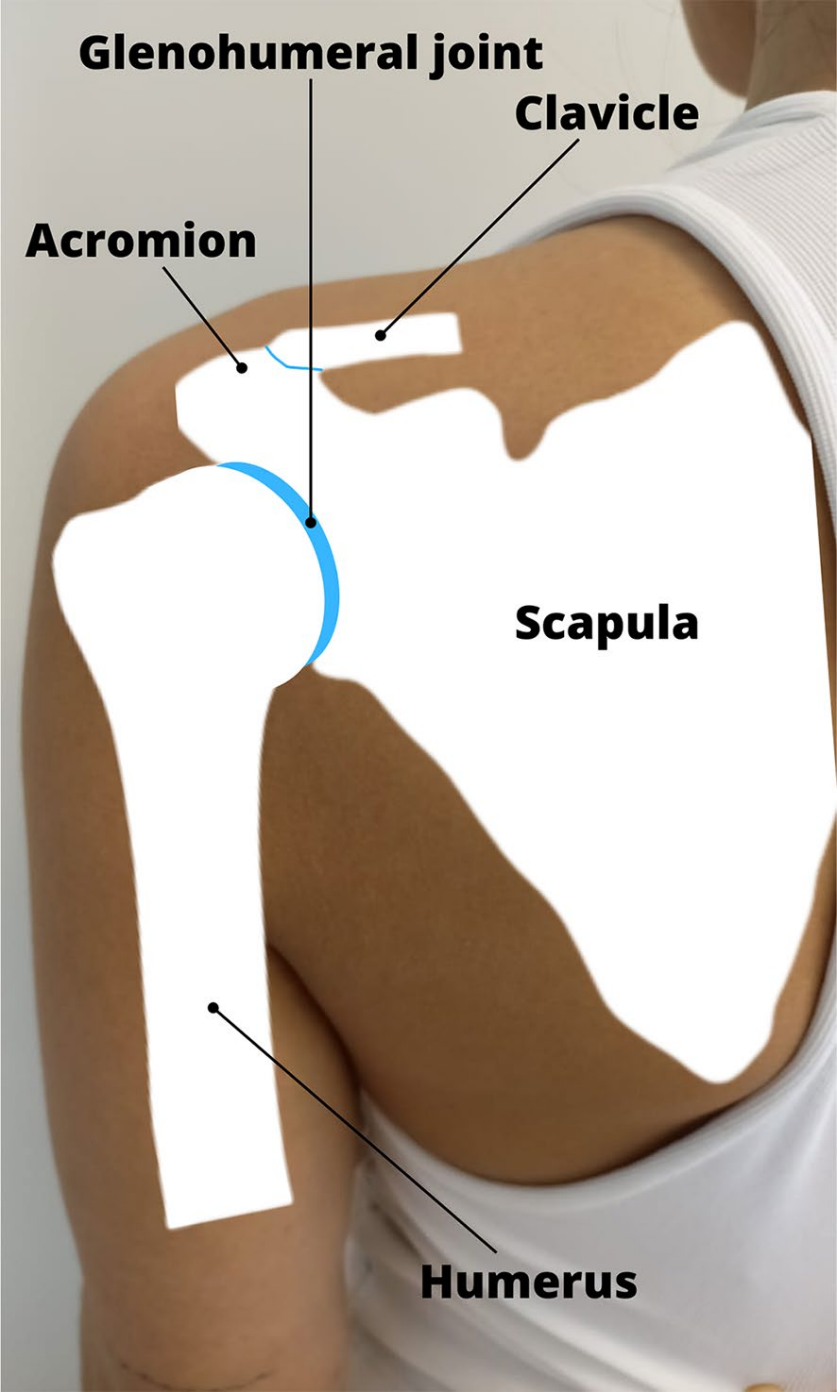
Anatomical conceptualization of the glenohumeral joint

For the rehabilitation of patients with neuromuscular pathologies, clinical professionals frequently resort to joint mobilization techniques or manual therapy aimed at maintaining or improving joint function and range [1–6]. A joint mobilization technique is defined as a technique that pursues the normalization of the global movement of the joint, preserving or improving its relation with the intimate displacement of the articular surfaces, and it does this through articulations of low velocity and high amplitude movements [7]. The glenohumeral joint is one of the most important joints in the shoulder and is formed by the connection of the humerus and the scapula **(**Fig. 1**)**. These types of techniques have been widely used in many very common shoulder pathologies, such as rotator cuff pathologies or shoulder impingements, not being the main therapeutic option in conservative treatment, but as one more tool that can contribute to the effect. of the intervention [8, 9]. The effects of manual therapy in diseases of glenohumeral joint have shown beneficial effects on pain and function, especially in the short term, and can be combined with other therapies such as exercise [10].

Manual therapy experts have been able to perfect the execution of the technique after years of experience repeating over again, which makes them have a good reliability when performing the technique contrary to the apprentices [11]. The learning process is divided in several cognitive and psychomotor phases: learning the procedure, seeing the demonstration, repeating the technique, identifying errors, correcting errors, correcting technique training and maintain the new skill [12]. This motor skill acquisition tried to capture several performance variables and change the way of movement and stabilization of the demanding task [13]. The demonstration of the teacher and the identification and immediate error correction of mistakes made by students is fundamental for the student to evolve in the learning process [14–16]. In addition, teachers should perceive improvements in the clinical applications, identify negative impact in the skills acquisition and give feedback to the students [17]. Traditional teaching methods are structured so that the teacher demonstrates the technique to be learned, the student performs and lastly learns it [16]. However, this teaching methodology has some limitations. First, the number of students who attend a class divided by the number of teachers or teacher-student ratio did not help the teacher to identify and correct errors. Moreover, the information that the student receives always depends on the subjectivity of the teacher [14, 18, 19].

Inertial sensors or inertial measurement units (IMU) provide real-time information on kinematic variables in the three axes of space. There is a research line that tries to demonstrate that the device gives a better acquisition of the technique according to the time, displacement and velocity during the teaching/learning process for techniques of high velocity and low amplitude manipulation, such as cervical, thoracic or ankle manipulations [19–21]. However, no studies have been found that use IMU as instruments that offer kinematic real-time feedback (KRTF) during the teaching/learning process of low velocity and high amplitude shoulder mobilization. Manual therapy is one of the most important ability in the clinical practice for physical therapist, thus, reducing the subjectivity of the teacher and correcting skill errors immediately influence during the process of acquiring a new motor skill [16, 22, 23]. The use of this instrument in manual therapy learning is important because it offers objective visual feedback in real-time following execution of the technique [24]. The visual feedback is useful also during the first phases of the acquisition of the manual therapy skills [24].

The main research question was “Can a new methodology based in KRTF improve the learning and synchronization of a manual therapy technique in the shoulder?”. The aim of the present study is to analyze the effectiveness of a KRTF, offered by an IMU, as a new methodology for learning a low velocity and high amplitude mobilization in the glenohumeral joint, comparing with the traditional teaching method. The main hypothesis was both groups improved the technique ability, but the KRTF learning methodology was more effective in the performance and the consistency than the traditional method for learning a mobilization of the glenohumeral joint.

## Methods

This section is devoted to explain the methodology used to carry out this study. In Sect. 2.1 and Sect. 2.2, the study design and participants are described respectively. Sections from 2.3 to 2.5 introduced the teaching methodologies, the technique description, and the experimental procedure. Finally, the statistical analysis was described in Sect. 2.6.

### Design

The present study is a randomized trial comparing the effect of a Kinematic Real-Time Feedback compared to the traditional teaching method for a shoulder mobilization technique. Recruitment and data collection were performed between 1 November 2016 and 31 April 2017 during the academic year. The study was conducted in the Universidad de Málaga (Malaga, Spain) as a teaching innovation project (PIE 15–24). The study obtained the clinicaltrials.gov registry number (NCT02504710) and followed the Consolidated Standards of Reporting Trials (CONSORT) guidelines [25].

### Participants

59 students from the Universidad de Málaga were part of the sample of the study. Students learned to perform the glenohumeral joint mobilization technique with two different types of teaching methodologies. The inclusion criterion used for all the participants in the present study was Bachelor of Physiotherapy degree students with no experience in manual therapy, so this technique was the first technique they learnt. The exclusion criteria used were experience in manual therapy (even in techniques other than the one selected for the present study), having undergone surgical intervention or fracture in shoulders or upper limbs in the year prior to the start of the study, and having suffered a dislocation of any joint of the body six months before the start of the study.

**Fig. 2:**
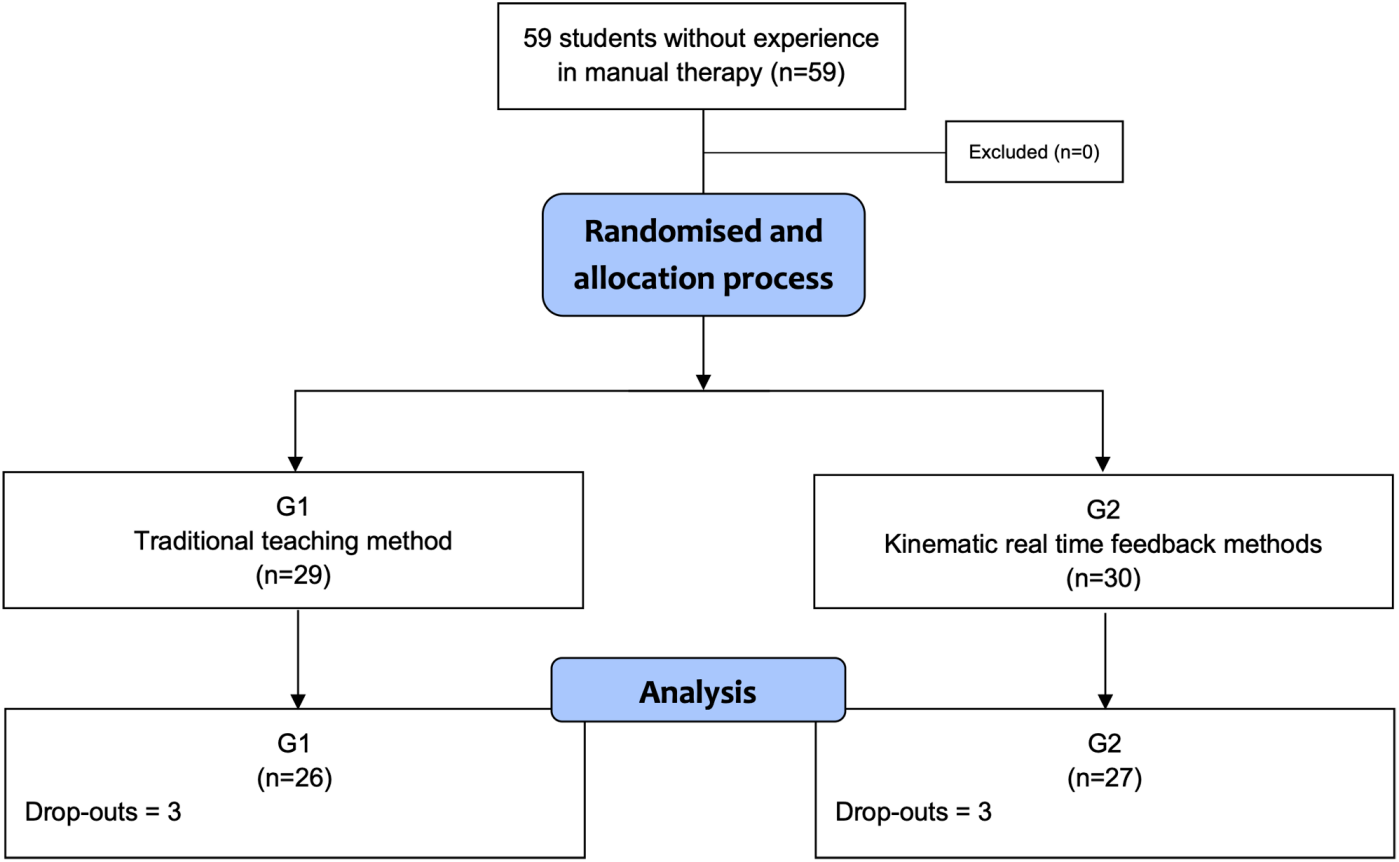
Flow of students through the trial

Students were randomized and balanced by gender using a software that generated random numbers and classified them into two different equal sized block groups (G1: Traditional methods group formed by 29 students; G2: KRTF group formed by 30 students). Three dropouts per group were found because the students did not attend to the second measurement (Fig. 2). Researchers contacted with these students to know and confirm the reason of the dropout and prevent possible bias due to his absence.

All participants included in the study had to give a signed informed consent. The study was approved by the Ethical Committee of the Universidad de Málaga (89-2015-H) and developed following the Ethical Principles for Medical Research Involving Human Subjects (Helsinki Declaration). The data were preserved and protected according to the Organic Law of Protection of Personal Data 15/1999.

### Instrument for kinematic real-time feedback

**Fig. 3:**
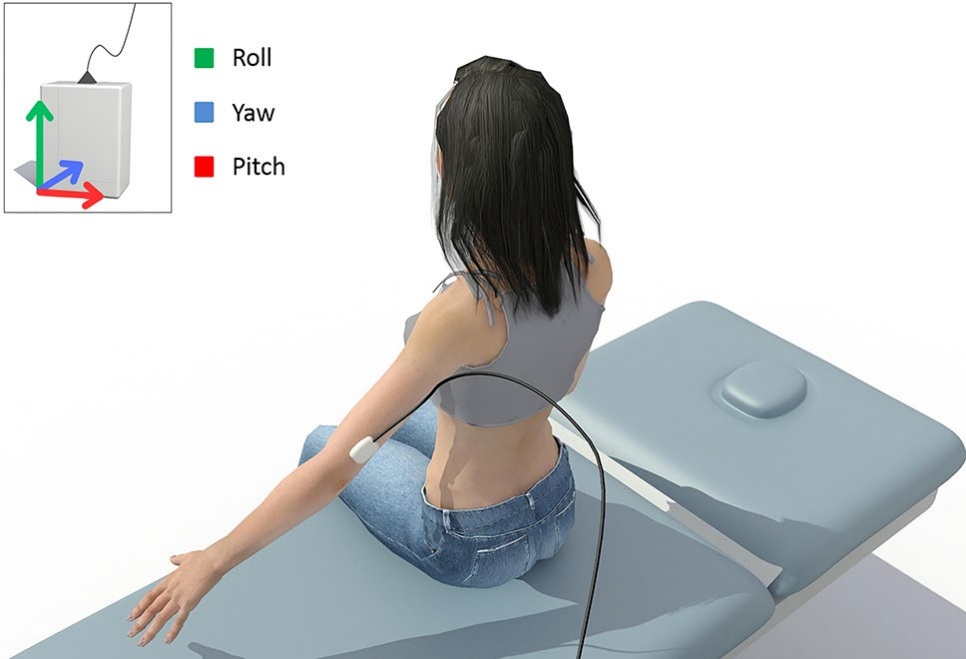
Placement and orientation of the IMU in the distal third of the forearm

An IMU (Inertial Cube 3, Intersense Inc., USA) with a sampling frequency of 180 Hz was used to perform the kinematic recording of the mobilization of the glenohumeral joint. The sensor was positioned on the posterior aspect of the distal third of the arm (Fig. 3). In addition, Fig. 3 shows the position of the sensor so that the origin of the coordinates (0,0,0 - Roll (X), Yaw (Y), and Pitch (Z), respectively) were in the leftmost postero-inferior vertex.

The fixation of the IMU to the skin was performed using a double-sided adhesive tape. It was also reinforced using a tape that surrounded the arm completely so that the sensor did not move with respect to the skin during the mobilization.

### Mobilisation technique

The glenohumeral joint mobilization was a passive mobilization for the patient. This low-velocity high-amplitude mobilization was performed out along available range of movement (grade II) according to the classification of the Maitland Concept manipulation discipline. This technique is useful in some shoulder pathologies when there is a lack of movement inside the joint because of tissue restrictions or inactivity so, the synchronization of these movements is an option of intervention in order to increase the range of movement and recover the joint. Two different movements were analyzed during the mobilization of the glenohumeral joint. First, the angulation movement is based on the functional movement of the shoulder in the frontal plane. There are two directions in this plane: abduction or moving the arm away from the central axis of the body and adduction or moving the arm towards the central axis of the body. The other movement, besides the angulation, is the translation or internal movement of the joint where the manual therapist makes an impulse to descent the humeral head with the hand [7].

**Fig. 4:**
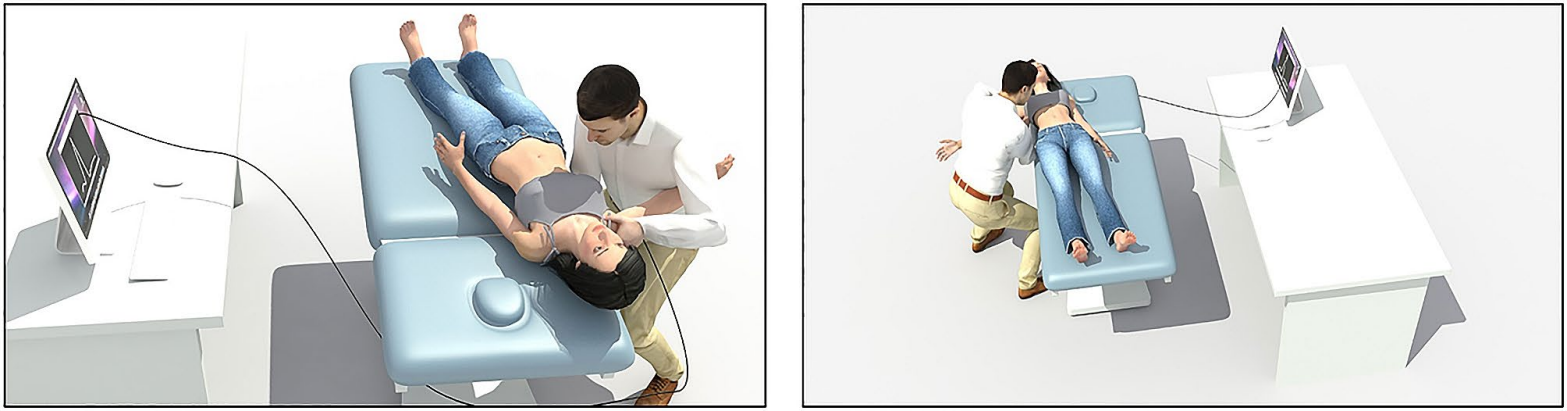
Scheme of the manual therapist and the patient lying on the stretcher during the execution of the glenohumeral joint mobilization with low speed and high amplitude

To perform the angulation movement, the manual therapist is placed in front of the stretcher, at the same level of the plane of the stretcher and on the homolateral side of the patient. It holds the patient’s arm from both humerus condyles with the arm and torso. The hands of the manual therapist were placed over the humeral head and at the lower angle of the scapula. The angulation movement is performed swinging from left to right and vice versa (changing the weight of the body from one leg to the other), causing one abduction and adduction movement on the patient’ glenohumeral joint. The translation movement is performed by the cranial hand over the humeral head causing a descent of it and favoring the joint mobilization. Figure 4 presents a schematic of the position of the manual therapist and the patient.

**Fig. 5:**
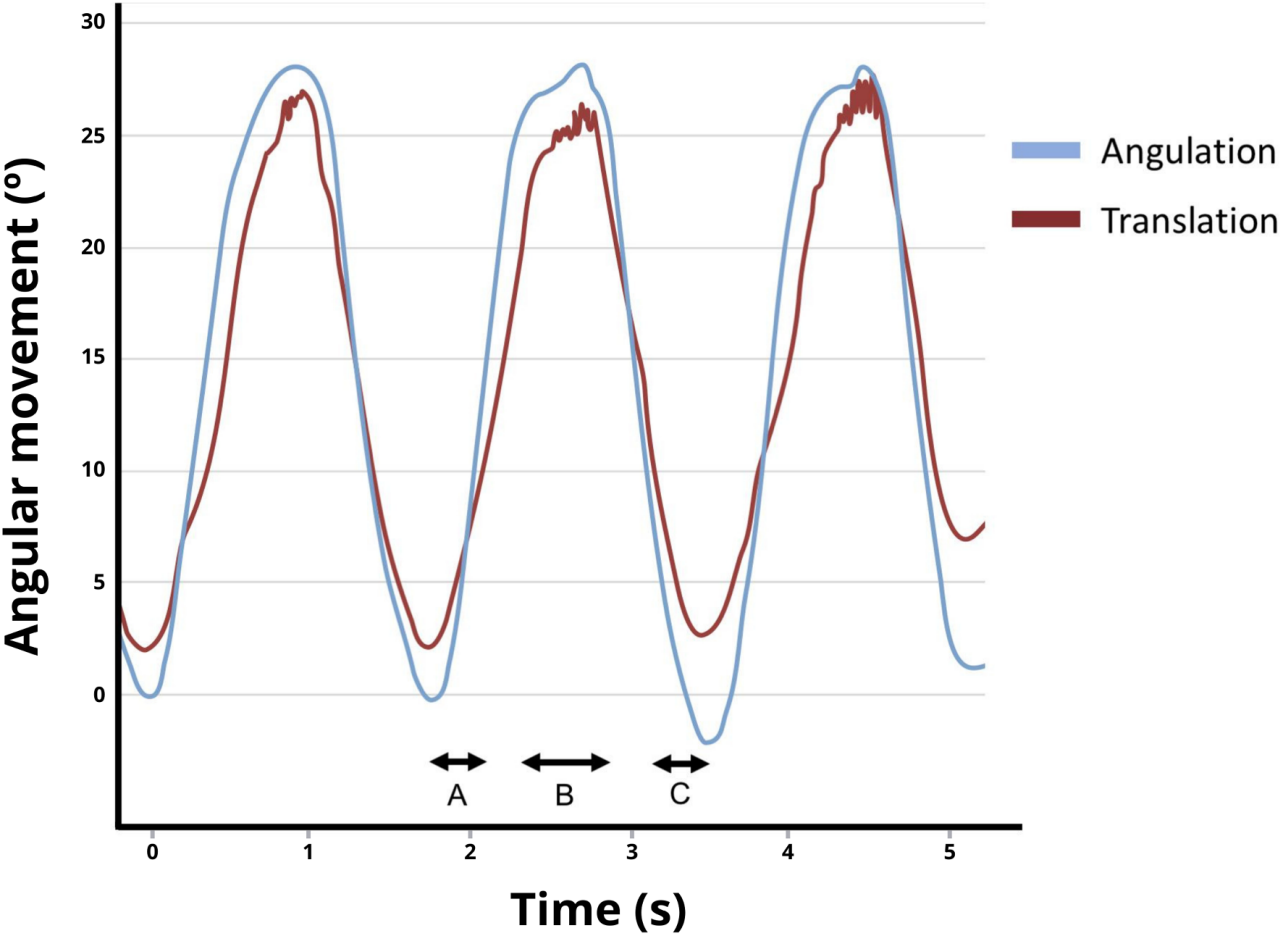
Typical angular movement for the glenohumeral joint mobilization using real-time kinematic feedback. A and C: The beginning and end of the Mobilization. B: The maximum arm abduction and descent of the humeral head

The complete mobilization integrates angulation and translation movements. When the manual therapist performs the abduction of the arm as previously explained, the cranial hand progressively performs a pressure toward the humeral head. When the manual therapist performs the abduction of the arm, returning to the starting position, this progressively removes pressure on the humeral head. These movements have a symmetrical performance so the start, increase and decrease of the angular displacement must be synchronized. Figure 5 shows an example of the mobilization with movement in this study. The standard mobilization includes angular movements and time recorded by the IMU in each repetition providing two similar curves in the graph.

To promote movement between the two articular surfaces (humeral head and glenoids), it is very important that, from the outer edge, the scapula is blocked in a neutral position during the complete development of the mobilization with the caudal hand [7]. The angulation movement was performed between 40° and 120° in order to give space for a good placement of the manual therapist and did not produce the natural beginning of the scapula movement.

### Protocol

The protocol of the present study was divided into two phases. In the first one, a teacher outside the study, with more than 20 years of experience in manual therapy, explained in detail the execution of the technique: first the angulation, then the translation and finally the integration of both movements (complete mobilization). Immediately afterwards, the teacher showed and explained a graph with the kinematic record of the mobilization to understand the complementary movements but G2 (KRTF method) learnt how to interpret both movements in the screen graph for an appropriate application.

When the explanation of the technique and the interpretation of the graph had been understood by the students, they made a first parameterized and collected repetitions (pre- measurement) recorded with the IMU that supposes the first record of the study. The sensor was placed according to the placement presented below and connected to a laptop. The kinematic information from the sensor was displayed in the screen in front of the student and collected using the app “ISPLOT” from the same provider as the sensor. The app created a file per each participant with the kinematic data that it was analyzed offline later. The data collector was carrying out the measurements, responsible to prepare the IMU in both before and after the intervention and saving the data. She was a blinded investigator with more than 10 years of experience in kinematic data analysis.

Next, the students practiced between themselves switching the role of manual therapist and model intensively for 90 min, following the standard dynamics of physiotherapy practices during the bachelor’s degree. Students only could practice strictly with members of their own group (G1 or G2). On one side, students in G1 (traditional method) practiced the technique explained while another manual therapy teacher supervised the practice of students by correcting the errors they make. The student: teacher ratio was 8:1. On the other hand, students in G2 are positioned at specific stations where they can perform the mobilization independently and observe the kinematic characteristics of the technique on a screen laptop. During the practice time, the student is alone and does not receive supervision from the teacher at any time.

The students used the upper right limb of the supposed patient if their dominant hand was the right and vice versa. After 90 min of practice, the second parameterized and collected repetitions (post- measurement) of the mobilization that was made.

During the parameterized execution of the mobilization (pre- and post- measurements) recorded by the IMU, each student performed 5 consecutive repetitions without stopping the mobilization. The second, third and fourth repetitions were chosen because students could improve the technique in the previous repetition, and they are not physically fatigued from repeating the repetitions yet.

### Statistical analyses

Sample size was calculated using the EPIDAT 3.1 program. A clinically significant effect (effect size d) of 0.8 would be of interest. Assuming that both groups started in the same condition and have no knowledge of the technique, with a two-sided significance of 0.025 and a power of 0.8, a total of 52 students (26 students per group) as minimum were required. In addition, an additional 10% was added in case of possible dropouts in the second measurement, thus, the final total sample was 58 participants.

All outcome variables were measured before and after the intervention. The change in yaw and roll from the initial position represented the two movements that compose the mobilization with movement (angulation and translation respectively). The pitch axis was not used because it did not matter in the mobilization technique.

For the two movements, the result variables extracted of both movements (angulation and translation) were: maximum angular displacement, minimum angular displacement, the difference between the maximum and the minimum angular displacement, area under the curve and the difference between the area under the curve. Besides the beginning and end of each mobilization, the calculation of the outcomes by MATLAB software (MathWorks, Natick, MA, USA) were identified.

**Fig. 6:**
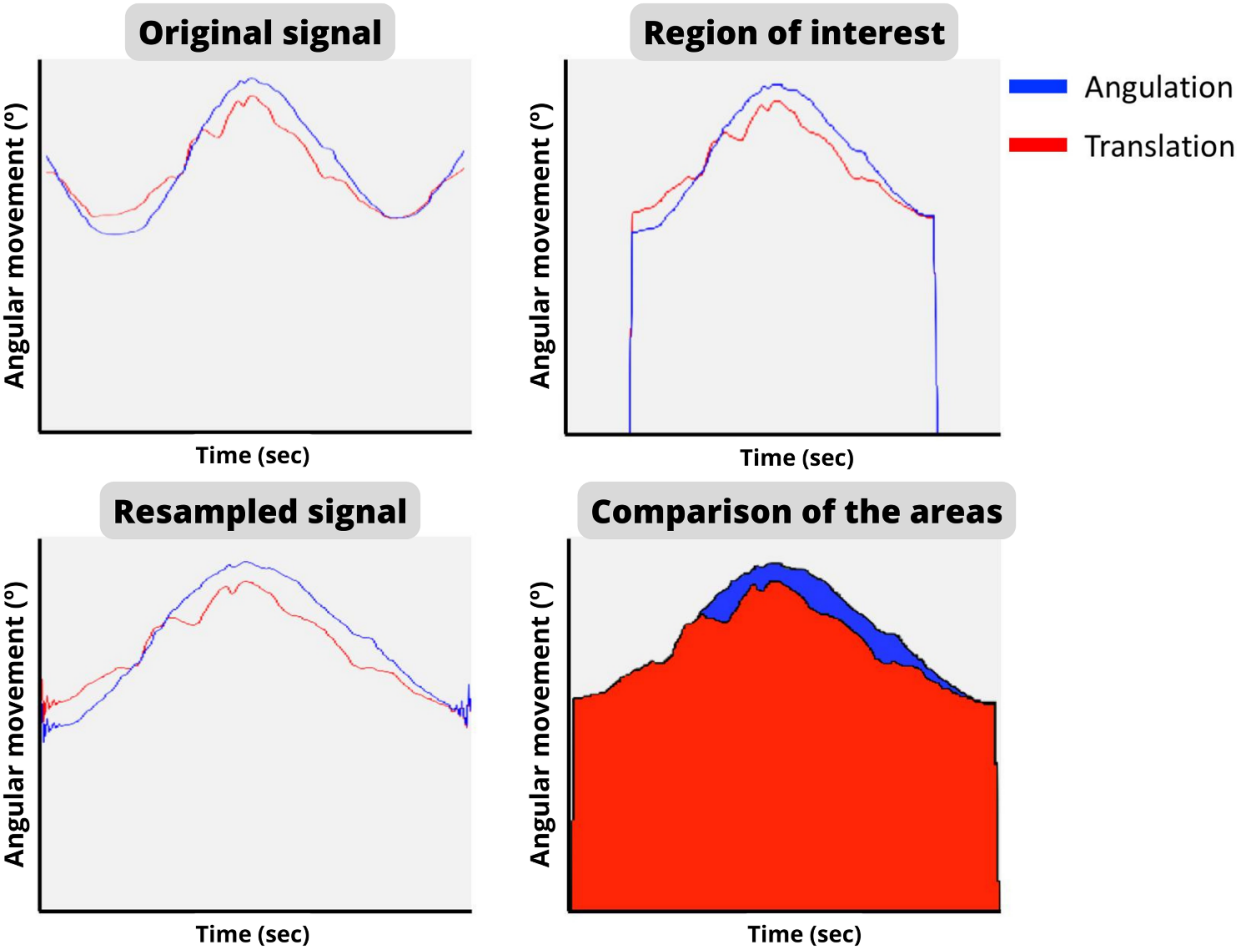
Evolution of the analysis of the data and graphs with MATLAB®. First, the original signal is processed to select the range of motion of one repetition. After that, the signal is resampled to a same time for comparing all the participant’s repetitions. Finally, the areas under the curve of each movement were compared

The area under the curve is the area created between the movement in a specific time inside a region of interest (ROI). Due to the y axis is the angular movement in degrees (°) and the x axis is the time in seconds (s), the unit of this outcome was Degrees x Seconds (°s). ROI was selected thanks to the delimitation of the same points in both curves using MATLAB. For the angular movement, the ROI was limited between the maximum peak of angulation displacement and 35° less than the maximum peak. For time (s), in order to be able to compare the outcome variables among the students, the mobilization that took the longest time was identified and all other mobilizations were resampled using the MATLAB function “resample()” (Fig. 6). The area under the curve was calculated by a numerical integration using the trapezoidal rule [26]. The MATLAB function “trapz()” was used for this purpose. Following the description of the manipulation previously described in Sect. 2.4., the angulation and translation areas must create a similar pattern. Then, a good execution of the technique was considered when the difference between areas is 0.

A descriptive analysis of the sample was performed to give more details about the characteristics of the students, which included basic anthropometric data (age, height, weight, and Body Mass Index (BMI)). An analysis of the distribution of the sample was performed using the Kolmogorov-Smirnoff test. An analysis of intra-group and inter-group differences was then performed for all outcome variables. For intra-group analysis (pre-post intervention), a one-way ANOVA was used and the Student’s t-test the inter-group analysis along to the 95% Confidence Interval (95% CI). In order to perform the group by time (Group x Time) interaction, a repeated measures ANOVA was used if the Mauchly’s sphericity test allowed to interpret these results. The level of significance was set at *p* ≤ 0.05.

In addition, an analysis of the internal consistency and reliability of all the outcome variables recorded during the mobilization was performed calculating Cronbach’s α and Intraclass correlation coefficient (ICC), respectively. Consistency and reliability analysis were performed with three consecutive mobilizations and results were stratified into different levels: excellent (value > 0.80), good (0.80 > value > 0.60), moderate (0.60 > value > 0.40) or poor (< 0.40) [27, 28]. In addition, a Bland-Altman plot was performed to show the agreement between the second and the fourth repetitions of the differences between areas (angulation and translation) in both methodologies in the post-intervention.

Statistical analysis was performed using the Statistical Package for Social Sciences (SPSS 21). The experimental study was performed by intention-to-treat analysis. The analysis of the data was done by a blinded and external investigator to study.

## Results

In total, 59 students participated in the present study. The G1 (traditional method) group consisted of 29 students (15 women/14 men) with the following anthropometric data: Average age: 21.98 (± 2.33) years; Height: 168.91 (± 9.71) cm; weight: 65.74 (± 8.01) kg, and BMI: 22.95 (± 2.97) kg/m^2^. The G2 (KRTF method) group consisted of 30 students (15 women/15 men) with the following anthropometric data: Mean age: 22.13 (± 2.46) Years, height: 167.19 (± 10.01) cm, weight: 66.08 (± 8.36) kg, and BMI: 23.35 (± 3.50) kg/m^2^. There were no significant differences between the groups in any of the anthropometric variables and in outcome variables measured in the baseline measure. Kolmogorov-Smirnoff and Mauchly’s sphericity tests confirmed the parametric distribution and the use of these results, respectively.

Table 1 presents the results comparing the outcome variables before and after the intervention, differences in the evolution of the components of each group after the intervention and the interaction between group and time. Four significant differences were found in the within group analysis in G2, as opposed to one significant difference in the G1. The within group analysis showed translation area were higher in G2 significantly (Mean difference = 1007.2°s) and the difference between areas was smaller in G2, too (G2 = 825.6°s). Only the difference between angulation and translation areas was significantly different in the group by time interaction (F = 4.14, *p* < 0.05).

**Table 1:** Study results from both groups. Mean differences within and between groups, and group x time interaction

						Within groups		Between groups	Group x Time interaction
		G1		G2		G1	G2		
		Pre (SD)	Post (SD)	Pre (SD)	Post (SD)	Mean difference (95% CI)	Mean difference (95% CI)	Mean difference (95% CI)	F
**Angulation**	Max Peak (°)	28.6 (15.8)	24.2 (12.7)	28.9 (14.5)	19.5 (9.5)	4.3 (-3.5, 12.2)	9.4 (2.9, 15.9)**	4.7 (-1.2, 10.8)	0.96
	Min Peak (°)	-4.9 (11.8)	-4.2 (9.4)	-5.2 (11.4)	-7.3 (7.5)	-0.6 (-5.0, 3.6)	2.1 (-3.1, 7.4)	3.0 (-1.4, 7.63)	0.73
	Dif Peak (°)	33.6 (19.0)	28.5 (13.6)	34.1 (18.5)	26.8 (11.5)	5.0 (-2.3, 12.5)	7.3 (-0.7, 15.3)	1.7 (-5.0, 8.45)	0.17
	Area (°s)	4857.6 (1437.9)	4815.4 (780.1)	5170.4 (10.8)	4721.3 (	42.2 (-498.9, 583.4)	449.1 (-77.7, 975.9)	94.1 (-354.6, 542.8)	1.18
**Translation**	Max Peak (°)	8.4 (10.9)	4.7 (7.7)	11.6 (11.4)	4.6 (7.3)	3.7 (-0.7, 8.1)	6.9 (1.5, 12.3)*	0.1 (-3.8, 4.11)	0.91
	Min Peak (°)	0.2 (9.3)	1.5 (6.0)	2.7 (11.6)	1.7 (9.7)	-1.2 (-5.7, 3.2)	1.0 (-4.4, 6.5)	-0.2 (-4.4, 3.9)	0.45
	Dif Peak (°)	8.2 (10.2)	3.2 (6.6)	8.8 (12.2)	2.9 (9.2)	4.9 (0.4, 9.4)*	5.9 (0.1, 11.7)*	0.3 (-3.8, 4.5)	0.06
	Area (°s)	2871.3 (2343.0)	2878.3 (1826.8)	3012.4 (2052.9)	3885.5 (1448.2)	-6.9 (-1108.0, 1094.1)	-873.1 (-1819.6, 73.4)	-1007.2 (-1889, -124.7)*	1.41
**Dif Areas (°s)**		2209.7 (1521.8)	1937.1 (1672.1)	2280.5 (1550.6)	825.6 (1148.2)	272.6 (-583.7, 1129.0)	1454.8 (662.9, 2246.6)***	1111.4 (338.3, 1884.5)**	4.14*

G1 (Traditional methods group), G2 (Kinematic Real-Time Feedback Method), Max Peak (Maximal Peak), Min Peak (Minimum peak), Dif Peak (Difference peak), Area (Area Under the Curve), Dif Area (Difference between areas: Angulation and translation)

Significance level: * *p* < 0.05; ** *p* ≤ 0.01; *** *p* ≤ 0.001

The consistency in all the variables in the G1 were between 0.90 and 0.99, and in the other group between 0.95 and 0.99. Regarding the reliability levels, G1 outcomes after the intervention were ranged between ICC = 0.76 and ICC = 0.97, while in the G2, the reliability levels in the outcome variables after the intervention ranged between ICC = 0.85 and ICC = 0.97. The complete internal consistency and reliability values in the post-intervention outcome variables can be observed in Table 2.

**Table 2:** Internal consistency and reliability analysis calculated through Cronbach’α and Intraclass Correlation Coefficient on both (new method and traditional method) groups post-intervention

		Angulation				Translation				
		Max peak	Min peak	Dif peak	Area	Max peak	Min peak	Dif peak	Area	Dif areas
**G1**	Cronbach’α	0.99	0.94	0.97	0.90	0.96	0.95	0.94	0.97	0.96
	ICC	0.97	0.84	0.93	0.76	0.91	0.88	0.85	0.93	0.88
**G2**	Cronbach’α	0.99	0.97	0.98	0.95	0.95	0.98	0.94	0.97	0.97
	ICC	0.97	0.92	0.95	0.86	0.86	0.96	0.85	0.92	0.91

G1 (Traditional methods group), G2 (Kinematic Real-Time Feedback Method), ICC (Intraclass Correlation Coefficient), Max Peak (Maximal Peak), Min Peak (Minimum peak), Dif Peak (Difference peak), Area (Area Under the Curve), Dif Area (Difference between areas: Angulation and translation)

**Fig. 7:**
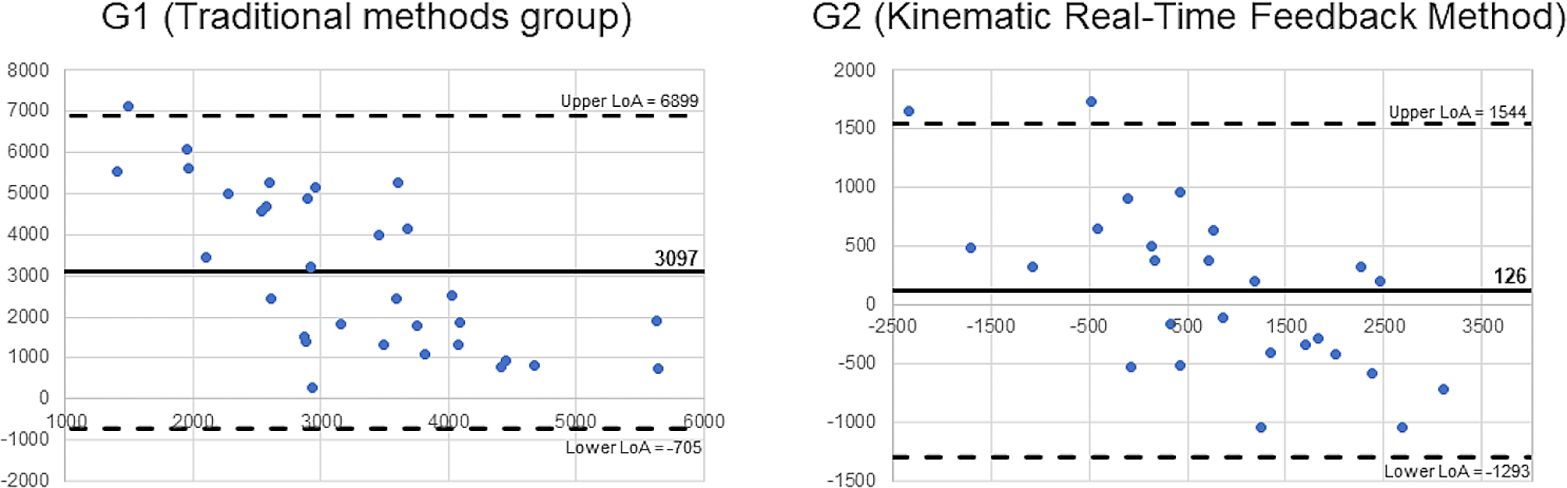
Bland-Altman plots of differences between areas in both groups in the post-intervention with the limits of agreement

Figure 7 showed the Bland-Altman plots from both groups in the differences between areas (angulation and translation).

## Discussion

To the best of our knowledge, this is the first study to analyze the effectiveness of a KRTF methodology, obtained through an IMU, as a new strategy for teaching/learning in students about the mobilization of glenohumeral joint compared to the traditional teaching methodology. The hypothesis of the authors was confirmed because G1 (traditional method) and G2 (KRTF method) showed significant differences in all outcome variables in the analysis but the G2 obtained less difference between areas, thus the effectiveness was better. Two groups experienced some changes in the mobilization of the glenohumeral joint but the significant difference in areas (angulation–translation) of G2 is closer to 0, which means that the execution of the technique was the closest thing to perfect. It is represented that it is very difficult to achieve the expert level in a lecture and the technique perfection should be obtained with the maintenance of the new skill [12]. Additionally, it has been observed that the execution consistency of the mobilization is superior in the G2 in comparison with G1.

### Within groups and between groups comparisons between methodologies

When comparing the results obtained before and after the learning period of the low velocity and high amplitude mobilization, it is possible to observe that several outcome variables presented significant differences in both intervention groups (Table 1). However, most of the important changes were described in G2. G2 students in the within groups comparison reduced the angulation (Mean Difference = 9.4°) and translation (Mean Difference = 6.9°) maximum peaks but they improved a lot the synchronization of both movements because translation area was higher after the intervention in G2 than G1 (Mean Difference = -1007.2°s). These results can be interpreted as these students made after the intervention a more controlled but precise techniques, controlling the range of the movements but make the same kinematics in time. These results are in accordance with other previous work that improved manual therapy learning using real-time feedback on lumbar spine joint mobilization [11]. On the other hand, G1 did significantly less translation in the peaks differences (Mean Difference = 4.9°) after the intervention but no more significant differences in within groups analysis. The technique of shoulder mobilization that has been used in the present study seeks to coordinate between the angulation and translation movements giving the same range of movement [7] and it seemed the G2 was closer to meeting that goal than the G1. Increased range of motion and displacement have also been observed previously in other joints, such as the ankle [19] and thoracic spine [20], among groups practicing manual therapy with real-time feedback using similar methodologies. It is clear that the learning of techniques where there is a motor skill to be developed is easier if there is a reliable method of feedback that quantifies the movement, parameterizes the performance and change possible mobilization errors [24, 29]. We cannot ignore the fact that the use of real-time feedback can lead the student to depend on having this tool to perform the technique well, explained by the guidance hypothesis [24]. It is necessary to perform more studies about low velocity and high amplitude techniques to prove the effectiveness and quantify the use to reduce dependency and improve long-term results.

One of the aims of the low velocity and high amplitude mobilization is to achieve the normal performance of the shoulder movement by improving the extensibility of the articular capsule. To reach this goal, it is necessary the coordination and synchronization of the translation and angulation movements in a specific range of motion [30, 31]. Regarding to the difference between the areas of both movements, it is considered a variable of special importance since it reports on the degree of simultaneity and symmetry between both movements. In G1, the difference in the areas after the intervention was more similar to the pre-measurement, whereas in the G2, the outcome was reduced to 825.6°s being the within group difference significantly (1454.8°s, *p* < 0.05) (Table 1). According to these results, the G2 performed a more coordinated mobilization after the learning because the difference in angulation and translation areas is closer to 0. Additionally, the between group difference between groups in the difference between areas was significant in the group x time interaction (F = 4.14). Thus, the movement of angulation and translation was more symmetric and simultaneous in G2 than G1. It was a challenge to analyze this technique because there are no other manual therapy learning studies that investigate the synchrony of two movements in a low velocity and high amplitude technique [24]. Other previous study also used the trapezoidal rule to obtain the area under the curve and compare changes in two different graphs [32]. The difference between the groups may be due to the fact that the low velocity and high amplitude mobilization used in the present study is a bimanual task [7]. The experts perform the bimanual task complex with very high levels of confidence, motor coordination and sensorial input [33], where coordination of trunk-limb movements is very important [34]. According to the G2 group had a higher difference between areas under the curve in both analysis, real-time feedback method has proven to be a solid method that improves skill levels and student autonomy in class reducing learning time.

### Consistency in the execution of the glenohumeral mobilisation

Consistency and reliability during performing a mobilization is very important to obtain the greatest benefit in the patient. When performing the technique repetitively, the good execution of the forces generated during the duration of the technique must be maintained. Keeping the good execution can differentiate an expert manual therapist from a student [22]. Prior to the intervention, consistency and reliability levels in outcome variables ranged from poor to moderate in both groups.

After the intervention, an excellent improvement in consistency and reliability was observed in all outcome variables in both groups, although the improvement was higher in G2 in Cronbach’s α and ICC analysis (0.95–0.99 and 0.85–0.97, respectively) than G1 (Cronbach’s α = 0.90–0.99 and ICC = 0.76–0.97) (Table 2) [28]. The main difference with the previous studies is that all of them have used the feedback system when learning the techniques of high velocity and low amplitude, while the present study analyzed a low velocity and high amplitude technique. The improvement in consistency performing shoulder mobilization is shown in line with previous studies where it was observed that the teaching methodology based on the KRTF obtained, in techniques of high velocity and low amplitude, levels of Cronbach’s α minimum consistency = 0.863 in manipulations of thoracic vertebrae [20] and in ankle mobilization (Cronbach’s α = 0.899) [19]. Comparing this time with manual therapy experts performing mobilization techniques, the students in G2 group achieved similar skills regarding consistency and reliability in cervical and lumbar mobilization than the experts [11, 21]. This similarity agreed with the new methodology is useful in the mobilization learning. In addition, the accuracy results showed similarities with previous studies that used instrumented treatment Table [22]. We can determine that consistency and reliability results are in line with other similar techniques and the same KRTF methodology and it can be very useful to improve the acquisition of the new motor skills thanks to the repeatability.

### Strengths and weaknesses

To the best of our knowledge, this is the first study where KRTF is used as a strategy for teaching/learning low velocity and high amplitude mobilization using an IMU. The mobilizations of low velocity and high amplitude require a high sensorimotor coordination of different body segments (upper limbs, trunk, lower limbs, etc.) for their correct execution. The IMU allows to decompose the movement into kinematic variables offering instant information and helping to improve the teaching/learning process of this mobilization. In addition, the IMU offers kinematic information in the three dimensions of space, a very important aspect when performing mobilization of the joint with greater degrees of freedom of the body. In addition, this type of learning strategy could be used in teaching/learning techniques that can be decomposed into kinematic variables, which are very common in the health professions (medicine, dentistry, podiatry, occupational therapy, etc.). The most relevant practical implication of this methodology is the objectification of the technique. This fact means that the student can better understand all the feedback that the teacher can give qualitative or subjective, in addition to the kinematic parameterization of the technique can create more objective evaluation standards that can accompany the subjective teaching rubric.

However, the present study presents some weaknesses, for example the no blinding of the students in the intervention. It was impossible to blind the students to two different learning techniques due to their great difference. Another limitation is the fact that students did the technique only using their dominant sides. There are manual therapy techniques in peripheral joints that require a change of the hand positions depending on the patients’ limb to be able to perform the manipulation. In this technique, it is necessary the hand position change but the non-dominant member not having been analyzed in this study. The change allows to the therapist to maintain the axis of force and reduce the complexity of the performance, but it is possible a decrease of the skill abilities or an asymmetric mobilization. We decided to use only dominant side to obtain more standardized results and allow to the student to perform the best manipulation they can do. It is important to have a both-sides learning because they should know how to mobilize the other side of the patient and this idea should be considered in future studies. In addition, other methodologies to assess the curves could be considered such as the comparison of the shapes of the curves.

Anthropometric differences between students can influence during practice time. This problem was minorized because the students practiced among themselves to recognize differences they may encounter with a real patient and from which they have to adapt. Additionally, the availability of teacher attention in G1 can also impair their learning process compared to the other group, so it was established a teacher: student ratio in 1:8 in order to watch all the mobilizations of the students.

The present study also analyzed the immediate outcome after the intervention, and it was an assumption of an expert performance of the technique in the initial term of the learning. Future studies could analyze the maintenance of learning in the medium and long-term in order to understand the complex psychomotor skills needed to automate the execution as undergraduate students. In addition, future studies could consider the learning theory “VAK (visual, auditory and kinesthetic)”, for which it would be necessary to expand the study sample. Next lines of research based on this study may be aimed at the use of new devices that increase immersion and distance learning such as immersive virtual reality, or kinematic studies that delve deeper into other parameters such as acceleration or speed.

## Conclusion

After comparing the kinematic variables recorded from students between two learning/teaching methodologies analyzed in the present study, we observed both methodologies improved after the learning period, but a better performance was showed in the KRTF group according to both movement in the pre-post measurements and comparing the area under the curve with the traditional methodology.

For the teaching/learning of the low velocity and high amplitude mobilization of the shoulder, KRTF is shown to be a very useful tool, as students without previous experience in manual therapy could significantly improve the capacity to perform this mobilization. The use of KRTF allows an increase in the autonomy of the student because the student received kinematic information on the execution of the mobilization to be able to modify those kinematic parameters allowing them to perform this skill correctly.

## Data Availability

The data is available contacting with the corresponding author by email.

## Abbreviations

IMU: Inertial measurement units
KRTF: Kinematic real-time feedback
G1: Traditional group
G2: KRTF group
ROI: Range of interest
BMI: Body mass index
ICC: Intraclass correlation coefficient
VAK: Visual, auditory and kinesthesic

## References

[1] An CM, Won JI. Effects of ankle joint mobilization with movement and weight-bearing exercise on knee strength, ankle range of motion, and gait velocity in patients with stroke: a pilot study. J Phys Ther Sci. 2016;28(2):689–94. 27065565 10.1589/jpts.28.689 PMC4793035

[2] Courtney CA, Steffen AD, Fernández-de-Las-Peñas C, Kim J, Chmell SJ. Joint mobilization enhances mechanisms of Conditioned Pain Modulation in individuals with osteoarthritis of the knee. J Orthop Sports Phys Ther. 2016;46(3):168–76. 26721229 10.2519/jospt.2016.6259

[3] Jang SH, Bang HS. Effect of thoracic and cervical joint mobilization on pulmonary function in stroke patients. J Phys Ther Sci. 2016;28(1):257–60. 26957769 10.1589/jpts.28.257 PMC4756015

[4] Mischke JJ, Emerson Kavchak AJ, Courtney CA. Effect of sternoclavicular joint mobilization on pain and function in a patient with massive supraspinatus tear. Physiother Theory Pract. 2016;32(2):153–8. 26863037 10.3109/09593985.2015.1114691

[5] Villafañe JH, Herrero P. Conservative treatment of myofascial trigger points and joint mobilization for management in patients with thumb carpometacarpal osteoarthritis. J Hand Ther. 2016;29(1):89–92. quiz 92. 26704595 10.1016/j.jht.2015.10.005

[6] Lantz JM, Emerson-Kavchak AJ, Mischke JJ, Courtney CA. Tibiofemoral joint mobilization in the successful management of patellofemoral pain syndrome: a case report. Int J Sports Phys Ther. 2016;11(3):450–61. 27274430 PMC4886812

[7] Cuesta-Vargas A, González-Sánchez M. Procedimientos básicos De Terapia Manual en El Sistema Neuro Miofascial (SNMF). Técnicas articulatorias in Métodos específicos de Intervención en fisioterapia (Sistema Músculo – Esquelético). Madrid: Ed Seco-Calvo J. Ed. Panamericana; 2015.

[8] Desjardins-Charbonneau A, Roy JS, Dionne CE, Frémont P, MacDermid JC, Desmeules F. The efficacy of manual therapy for rotator cuff tendinopathy: a systematic review and meta-analysis. J Orthop Sports Phys Ther. 2015;45(5):330–50. 25808530 10.2519/jospt.2015.5455

[9] Innocenti T, Ristori D, Miele S, Testa M. The management of shoulder impingement and related disorders: a systematic review on diagnostic accuracy of physical tests and manual therapy efficacy. J Bodyw Mov Ther. 2019;23(3):604–18. 31563378 10.1016/j.jbmt.2018.08.002

[10] Steuri R, Sattelmayer M, Elsig S, Kolly C, Tal A, Taeymans J, et al. Effectiveness of conservative interventions including exercise, manual therapy and medical management in adults with shoulder impingement: a systematic review and meta-analysis of RCTs. Br J Sports Med. 2017;51(18):1340–7. 28630217 10.1136/bjsports-2016-096515 PMC5574390

[11] Petersen EJ, Thurmond SM, Shaw CA, Miller KN, Lee TW, Koborsi JA. Reliability and accuracy of an expert physical therapist as a reference standard for a manual therapy joint mobilization trial. J Man Manip Ther. 2021;29(3):189–95. 33234048 10.1080/10669817.2020.1844853 PMC8183505

[12] Sawyer T, White M, Zaveri P, Chang T, Ades A, French H, et al. Learn, see, practice, prove, do, maintain: an evidence-based pedagogical framework for procedural skill training in medicine. Acad Med. 2015;90(8):1025–33. 25881645 10.1097/ACM.0000000000000734

[13] Pacheco MM, Lafe CW, Newell KM. Search Strategies in the Perceptual-Motor Workspace and the Acquisition of Coordination, Control, and Skill. Frontiers in Psychology [Internet]. 2019 [cited 2023 Feb 27];10. https://www.frontiersin.org/articles/10.3389/fpsyg.2019.01874. 10.3389/fpsyg.2019.01874 PMC6702327 31474912

[14] Schmidt RA, Zelaznik H, Hawkins B, Frank JS, Quinn JT. Motor-output variability: a theory for the accuracy of rapid motor acts. Psychol Rev. 1979;47(5):415–51. 504536 10.1037/0033-295X.86.5.415

[15] Enebo B, Sherwood D. Experience and practice organization in learning a simulated high-velocity low-amplitude task. J Manipulative Physiol Ther. 2005;28(1):33–43. 15726033 10.1016/j.jmpt.2004.12.002

[16] Nicholls D, Sweet L, Muller A, Hyett J. Teaching psychomotor skills in the twenty-first century: revisiting and reviewing instructional approaches through the lens of contemporary literature. Med Teach. 2016;38(10):1056–63. 27023405 10.3109/0142159X.2016.1150984

[17] de Luca K, McDonald M, Montgomery L, Sharp S, Young A, Vella S, et al. COVID-19: how has a global pandemic changed manual therapy technique education in chiropractic programs around the world? Chiropr Man Th. 2021;29(1):7. 10.1186/s12998-021-00364-7 PMC7849220 33522933

[18] Flynn D, Eddy ER, Tannenbaum SI. The impact of National Culture on the continuous learning environment. J East-West Business. 2006;12(2–3):85–107. 10.1300/J097v12n02_05

[19] González-Sánchez M, Ruiz-Muñoz M, Ávila-Bolívar AB, Cuesta-Vargas AI. Kinematic real-time feedback is more effective than traditional teaching method in learning ankle joint mobilisation: a randomised controlled trial. BMC Med Educ. 2016;16(1):261. 27716215 10.1186/s12909-016-0789-8 PMC5054622

[20] Cuesta-Vargas AI, González-Sánchez M, Lenfant Y. Inertial sensors as real-time feedback improve learning posterior-anterior thoracic manipulation: a randomized controlled trial. J Manipulative Physiol Ther. 2015;38(6):425–33. 26215901 10.1016/j.jmpt.2015.04.004

[21] Cuesta-Vargas AI, Williams J. Inertial sensor real-time feedback enhances the learning of cervical spine manipulation: a prospective study. BMC Med Educ. 2014;14:120. 24942483 10.1186/1472-6920-14-120 PMC4075507

[22] Snodgrass SJ, Odelli RA. Objective concurrent feedback on force parameters improves performance of lumbar mobilisation, but skill retention declines rapidly. Physiotherapy. 2012;98(1):47–56. 22265385 10.1016/j.physio.2011.02.002

[23] Wajon A, Ada L, Refshauge K. Work-related thumb pain in physiotherapists is associated with thumb alignment during performance of PA pressures. Man Ther. 2007;12(1):12–6. 16843032 10.1016/j.math.2005.09.003

[24] Chang I, Rivera MJ, Eberman LE. The Effect of Feedback on Manual Therapy Skill Acquisition: a systematic review. Athletic Train Educ J. 2020;15(3):224–34. 10.4085/150120038

[25] Schulz KF, Altman DG, Moher D, the CONSORT Group. CONSORT 2010 Statement: updated guidelines for reporting parallel group randomised trials. BMC Med. 2010;8(1):18. 20334633 10.1186/1741-7015-8-18 PMC2860339

[26] Lallemand M, Giboreau A, Rytz A, Colas B. Extracting parameters from Time-Intensity curves using a trapezoid model: the Example of some sensory attributes of ice cream. J Sens Stud. 1999;14(4):387–99. 10.1111/j.1745-459X.1999.tb00123.x

[27] Portney L, Watkins M. Foundations of clinical research. 2nd ed. Upper Saddle River, NJ: Pearson/Prentice Hall; 1999.

[28] Shrout PE, Fleiss JL. Intraclass correlations: uses in assessing rater reliability. Psychol Bull. 1979;86(2):420–8. 18839484 10.1037/0033-2909.86.2.420

[29] Zhang X, Shan G, Wang Y, Wan B, Li H, Wearables. Biomechanical Feedback, and Human Motor-skills’ Learning & optimization. Appl Sci. 2019;9(2):226. 10.3390/app9020226

[30] Noten S, Meeus M, Stassijns G, Van Glabbeek F, Verborgt O, Struyf F. Efficacy of different types of mobilization techniques in patients with primary Adhesive Capsulitis of the shoulder: a systematic review. Arch Phys Med Rehabil. 2016;97(5):815–25. 26284892 10.1016/j.apmr.2015.07.025

[31] Hengeveld E, Hengeveld KB E, Banks KB. (2014) Maitland’s peripheral Manipulation - Management of Neuromusculoskeletal Disorders - Vol II. 5th ed. 2014.

[32] Chitupe A, Joshi S. Classification of ECG Data for Predictive Analysis to assist in medical decisions. IJCSNS Int J Comput Sci Netw Secur. 2015;15(10):48–53.

[33] Knobe M, Holschen M, Mooij SC, Sellei RM, Münker R, Antony P, et al. Knowledge transfer of spinal manipulation skills by student-teachers: a randomised controlled trial. Eur Spine J. 2012;21(5):992–8. 22223196 10.1007/s00586-011-2140-8 PMC3337919

[34] Wulf G, McNevin NH, Fuchs T, Ritter F, Toole T. Attentional focus in complex skill learning. Res Q Exerc Sport. 2000;71(3):229–39. 10999260 10.1080/02701367.2000.10608903

